# Peripartum allopregnanolone blood concentrations and depressive symptoms: a systematic review and individual participant data meta-analysis

**DOI:** 10.1038/s41380-024-02747-7

**Published:** 2024-11-07

**Authors:** Georgios Schoretsanitis, Lauren M. Osborne, Inger Sundström-Poromaa, Elizabeth S. Wenzel, Jennifer L. Payne, Corrado Barbui, Chiara Gastaldon, Kristina M. Deligiannidis

**Affiliations:** 1 Northwell Health, Department of Psychiatry, the Zucker Hillside Hospital, Glen Oaks, NY USA; 2 Department of Psychiatry, Psychotherapy and Psychosomatics, Hospital of Psychiatry, University of Zurich, Zurich, Switzerland; 3 Departments of Obstetrics and Gynecology and of Psychiatry, Weill Cornell Medical College, New York, NY USA; 4 Department of Women’s and Children’s Health, Uppsala University, Uppsala, Sweden; 5 Department of Psychology, University of Illinois, Chicago, IL USA; 6 Department of Psychiatry and Neurobehavioral Sciences, University of Virginia, Charlottesville, VA USA; 7 WHO Collaborating Centre for Research and Training in Mental Health and Service Evaluation, Department of Neuroscience, Biomedicine and Movement Sciences, Section of Psychiatry, University of Verona, Verona, Italy; 8 Institute for Social and Preventive Medicine, University of Bern, Bern, Switzerland; 9 Departments of Psychiatry and Obstetrics & Gynecology, Donald and Barbara Zucker School of Medicine at Hofstra/Northwell, Hempstead, NY USA; 10 Institute for Behavioral Sciences, Feinstein Institutes for Medical Research, Manhasset, NY USA

**Keywords:** Predictive markers, Depression

## Abstract

Neuroactive steroids including allopregnanolone are implicated in the pathophysiology of peripartum depressive symptoms (PDS). We performed a systematic review searching PubMed/Embase/PsychInfo/Cinhail through 08/2023 (updated in 07/2024), and conducted a random-effects meta-analysis of studies comparing allopregnanolone blood concentrations in women with versus without PDS at various timepoints during the 2^nd^ and 3^rd^ trimester and the postpartum period, calculating standardized mean differences (SMDs) and 95% confidence intervals (CIs). Meta-regression and subgroup analyses included age, diagnoses of affective disorders before pregnancy, antidepressant treatment, analytical methods, and sample type. Study quality was assessed using the Newcastle-Ottawa-scale. The study protocol was registered on PROSPERO (registration number CRD42022354495). We retrieved 13 studies with 2509 women (*n* = 849 with PDS). Allopregnanolone concentrations did not differ between women with versus without PDS at any timepoint (*p* > 0.05). Allopregnanolone concentrations assessed during pregnancy did not differ for women with versus without PDS at postpartum follow-up (*p* > 0.05). Subgroup analyses indicated higher allopregnanolone concentrations in women with versus without PDS at gestational weeks 21–24 and 25–28 (SMD = 1.07, 95% CI = 0.04, 2.11 and SMD = 0.92, 95% CI = 0.26, 1.59 respectively). Moreover, we reported differences between studies using mass-spectrometry combined with chromatography versus immunoassays at gestational weeks 25–28 (*p* = 0.01) and plasma versus serum samples at gestational weeks 21–24 (*p* = 0.005). Study quality was rated as poor, good, and fair for two, one and ten studies respectively. PDS were not associated with differences for allopregnanolone concentrations. The use of heterogenous peripartum time points, study cohorts, depression symptom measures and analytical methods has hampered progress in elucidating neuroactive steroid signaling linked to PDS.

## Introduction

Depression arising in pregnancy or the postpartum period (together referred to as peripartum depression) is the most common complication of childbirth and a major preventable cause of maternal mortality [1, 2]. The prevalence varies according to population, risk factors, and time of onset, but in most populations reaches at least 15–20% [3, 4]. Untreated peripartum depression can have short- and long-term harmful consequences for mother and offspring, including decreased maternal functioning [5], maternal-infant bonding difficulties [6], lactation failure [7] and impaired cognitive, behavioral and emotional development of the child [8–10].

Peripartum depression is a reproductive mood disorder, one of several depressive disorders which are hypothesized to be in part triggered by sensitivity to reproductive and stress-related steroids during reproductive transitions. The evidence supports a multifactorial mechanism of disease hypothesis involving the integration of psychosocial and biological risk factors including genetic, epigenetic, synaptic transmission, immune and endocrine factors [11]. One aspect of this hypothesis is that patients susceptible to peripartum depression have a higher sensitivity to stress during phases of neuroactive steroid fluctuation in pregnancy and/or the postpartum period [11]. This sensitivity may correspond to altered allopregnanolone-modulating functioning at the gamma-aminobutyric acid (GABA-A-R) within the stress circuitry [12].

Neuroactive steroids, including allopregnanolone (3α, 5α-tetrahydroprogesterone), are endogenously synthesized from cholesterol or exogenous synthetic steroids that exert actions upon the brain. Several neuroactive steroids are positive allosteric modulators (PAMs) of the inhibitory GABA-A-R, the ligand-gated and membrane-bound pentameric ion channels that mediate passage of negatively charged chloride ions into the post-synaptic membrane [13–15]. Synaptically located GABA-A-Rs contribute to low-affinity phasic inhibition while extrasynaptically located GABA-A-Rs contribute to high affinity tonic inhibition, ultimately leading to changes in the excitatory–inhibitory balance of the brain networks in which they are located and functionally connected [16, 17]. Neurocircuit modulators, neuroactive steroids are critical in the regulation of the hypothalamic–pituitary–adrenal (HPA) axis during acute and chronic stress as well as nonstress conditions [18, 19] and new evidence suggests that they may set a baseline affective tone, one that is impacted by risk factors for psychiatric illnesses [20]. Preclinical models suggest that allopregnanolone specifically acts as a local inhibitor and provides a long-loop negative feedback to the HPA axis [21]. Preclinical evidence also suggest that allopregnanolone may be synthesized in the brain independently of the adrenal glands [22].

Allopregnanolone and its analogs have been shown to be effective in the treatment of severe postpartum depression [23–28], yet allopregnanolone’s role in its pathophysiology has been challenging to elucidate. Despite decades of neuroactive steroid research, a consistent association between neuroactive steroid blood or CNS levels and PDS has been elusive [29–33], despite robust data on their pathophysiological role in preclinical models [12, 34, 35]. For example, the first human study including cross-sectional allopregnanolone concentrations measurements in women with postpartum depressive symptoms reported lower allopregnanolone compared to euthymic women early after delivery [30]. The finding of lower allopregnanolone blood concentrations in women with postpartum depressive symptoms was not replicated in later studies [32, 33, 36, 37]. In contrast, another study suggested a positive association between allopregnanolone concentrations and depression severity at various peripartum timepoints [38].

Overall, study designs differ in their selection of peripartum timepoints and symptom measures for analysis and neuroactive steroid analytic methods (e.g. immunoassays, mass spectrometry, etc.). Additionally, previous studies examined a variety of clinical phenotypes, ranging from women at risk for peripartum depression to those with active antepartum or postpartum depression or those with subclinical postpartum blues. Timing of neuroactive steroid measurements in relationship to symptom onset has differed as well. Given that some psychotropics may affect peripheral neuroactive steroids levels [39, 40], in addition to brain concentrations [20], some studies have excluded the use of psychotropic agents, however others have not. Thus, knowledge regarding allopregnanolone concentration patterns and PDS remains fragmented, despite advances in the field with recent transformative US Food & Drug Administration (FDA)-approvals of brexanolone and zuranolone, two neuroactive steroid-based pharmacotherapies for postpartum depression.

Our aim therefore was to systematically review and subsequently meta-analyze observational data on blood measures of allopregnanolone in peripartum women with depressive symptoms compared to women without depressive symptoms and assess potential moderators of allopregnanolone patterns.

## Methods and materials

The study was conducted with use of MOOSE guidelines for meta-analyses of observational studies [41] and was registered with PROSPERO (registration number CRD42022354495). We identified observational studies measuring peripheral (serum or plasma) allopregnanolone concentrations in peripartum women and also including assessments of mood symptoms by searching Medline and Embase, using the following search terms: (allopregnanolone OR 3α, 5α-tetrahydroprogesterone) AND (postpartum OR postnat* OR antenat* OR peripartum OR pregnan* OR perinatal) AND (depress* OR affective) AND (blood OR serum OR plasma). An additional search in PsychInfo and Cinhail was performed. Databases were searched last on August 1^st^, 2023, since data inception without language restrictions. We updated the search in July 2024 without identifying new studies of interest. Subsequently, references from identified studies were hand-searched for additional works of potential interest.

### Inclusion & exclusion criteria

#### Type of studies

We included studies reporting on allopregnanolone blood concentrations in women with versus without depressive symptoms during the peripartum period, regardless of the treatment setting. We selected depressive symptoms (instead of clinical diagnoses) aiming for more precision given the dynamic nature of symptoms during the peripartum period.

#### Types of participants

Women with versus without depressive symptoms during the peripartum period were included. There were no restrictions with regards to treatment setting or symptom duration. Participants included antidepressant-naïve and antidepressant-free women as well as those receiving psychotropic treatments. For the sake of fluency, throughout this paper we will refer to the birthing individuals who participated in these studies as “female,” “women,” and “mothers,” while acknowledging that not all individuals identify with these labels.

#### Types of exposure

Depressive symptoms at any time during pregnancy and during postpartum up to one year after delivery.

## Outcomes

The primary outcome was defined as differences in mean blood concentrations of allopregnanolone between women with versus without depressive symptoms across different peripartum timepoints. For studies using multiple depressive symptom measures, we completed our analysis using the Edinburgh Postnatal Depression Scale (EPDS) [42], to reduce heterogeneity between studies. As the blood concentrations vary greatly over the peripartum period [43, 44], we considered eight different timepoints. Specifically, we considered 4-week intervals during pregnancy: a) 1^st^ trimester, <12 week, b) 2^nd^ trimester, 12–16 weeks, c) 2^nd^ trimester, 17–20 weeks, d) 2^nd^ trimester, 21–24 weeks, e) 3^rd^ trimester, 25–28 weeks, f) 3^rd^ trimester, 29–33 weeks, g) 3^rd^ trimester, ≥34 week and two timepoints at postpartum h) ≤1 week after delivery and i) at postpartum ≥2 weeks.

### Screening and data extraction

Two authors (CG and GS) independently selected studies of interest. No additional co-author was involved as consensus was reached for all cases.

Two authors (CG and GS) independently extracted data regarding sample sizes, demographic characteristics, ratings of depressive symptoms, analytical method and timepoint of allopregnanolone assessment, types of allopregnanolone blood samples, and blood allopregnanolone concentrations (mean and standard deviation [SD]). Before data entry, allopregnanolone concentrations were converted to the same unit (nmol/L to ng/mL) and weighted means for covariates were computed based on means of subgroups. When required, authors were contacted to provide details or raw data from their studies.

### Quality of studies

The modified version of the Newcastle-Ottawa scale for cohort and cross-sectional studies was used for quality assessment [45]; we removed the item “representativeness of the exposed cohort” which we judged to be related to applicability, and added ascertainment of ratings of postpartum depressive symptoms as described elsewhere [46].

### Statistical analysis

We used a random-effects model for our primary outcome, considering the large heterogeneity related to study cohorts, analytical methods, and the inherently essential related variability. Results for each timepoint of allopregnanolone assessments were summarized using the standardized mean difference (SMD) and 95% confidence intervals (CI) presented in forest plots; further, we assessed the predictive role of gestational allopregnanolone concentrations’ differences between women with versus without depressive symptoms at postpartum follow-ups. The heterogeneity variance parameter (*τ*^2^) was calculated using the DerSimonian-Laird estimator [47]. For longitudinal studies with allopregnanolone assessments at multiple timepoints, cohorts from the same study were considered separately for different meta-analyses in a cross-sectional manner, based on the eight predefined timepoints. We also calculated the I-square (*I*^2^) statistic as a measure of the proportion of variability that can be attributed to heterogeneity [48]. Thereafter, the effect of maternal age was assessed in a meta-regression analysis [49]. Subgroup analyses included cohorts with patients with affective disorders prior to pregnancy, with patients treated with antidepressants during study period, different analytical methods and sample types (comparing patterns in cohorts using plasma versus serum samples); the latter was performed as measurement of progesterone (and progesterone derivates) may be very sensitive to pre-analytic variables [50]. We also performed a sensitivity analysis excluding poor-quality studies. Meta-regression and subgroup analyses were performed when data for a minimum of three studies were available. Last, we assessed the publication bias inspecting funnel plots and performing Egger’s test when at least 10 studies were available [51]. Analyses were performed using the meta package in R [52].

## Results

The electronic database search yielded 203 articles from Pubmed/Medline and 94 from Embase, whereas the additional search in PsychInfo and Cinhail did not report any additional relevant studies. After removing 24 duplicates, 273 unique articles remained. After exclusion of 241 articles based on title and abstract review, 32 full-text articles were screened, leading to exclusion of 21 papers due to works/posters with overlapping data (*n* = 10), reviews (*n* = 5), effects of interventions on allopregnanolone concentrations (*n* = 3), no peripartum assessments (*n* = 2), and lack of stratified allopregnanolone concentrations for women with versus without peripartum depressive symptoms (*n* = 1). A search update in July 2024 yielded three more works, one of which was a systematic review (and was excluded) and two works that were included. Ultimately, thirteen studies fulfilled all inclusion criteria and were included in our systematic review and meta-analysis (Supplementary Fig. 1).

### Quality assessment

Out of the thirteen studies included for the primary outcome, ten were rated as fair, two as poor, and one as poor quality (Supplementary Table 1). Quality issues mainly included lack of power analysis and/or lack of specification for assessment of allopregnanolone concentrations blinded to mood symptom ratings.

### Study and patient characteristics

We meta-analyzed eleven studies with 2509 women (mean age = 27.9 ± 5.8 years) including 849 with peripartum depressive symptoms, who were compared with 1660 women without peripartum depressive symptoms. One study additionally included ten non-peripartum healthy controls (Table 1).

**Table 1:** Characteristics of included studies (in chronological order).

Author, year	Total subjects	Group	*n*	Maternal age (SD), years	Diagnosis of affective disorders before pregnancy	Treatment with AD	Allopregnanolone levels’ assessment				Mood symptoms scales		Quality
							Blood levels (SD), ng/mL	Timepoint (SD)	Method	Sample type	Scales	Cut-off^a^	
Nappi, 2001	40	PDS	18	31.0 (4.3)	0 (0.0)	0 (0.0)	0.34 (0.12)	PP 3 days	RIA	Serum	HDRS-17/SQ	11–18	Poor
		Not depressed	22	29.8 (2.9)			0.72 (0.31)						
Pearson Murphy, 2001	68^b^	Depressed	9	21–45	0 (0.0)	0 (0.0)	3.67 (0.72)	GW 26–28	HPLC	Plasma	HDRS	13	Fair
		Not depressed	12				2.92 (1.04)						
		Depressed	6				4.18 (1.10)	GW 36–38					
		Not depressed	9				4.24 (1.82)						
		Depressed	5				0.75 (0.24)	PP 2–7 days					
		Not depressed	11				0.92 (0.61)						
		Depressed	5				0.32 (0.25)	PP 13–18 days					
		Not depressed	11				0.24 (0.12)						
Epperson, 2006	33	Depressed	9	30.0 (5.3)	4 (44.4)	4 (44.4)	0.25 (0.12)^c^	PP < 24 weeks	GC/MS	Plasma	HDRS-19	18	Fair
		Healthy controls	14	31.0 (2.9)	0 (0.0)	0 (0.0)	0.21 (0.07)^c^						
		non-peripartum healthy controls	10	28.7 (3.8)	0 (0.0)	0 (0.0)	0.50 (0.20)	NA					
Paoletti, 2006	14	Depressed	7	31.8 (3.2)	0 (0.0)	0 (0.0)	8.2 (0.6)	GW 14	RIA	Serum	SCL-90	50	Poor
							10.8 (0.7)	GW 22					
							16.8 (0.8)	GW 30					
							18.1 (1.1)	GW 40					
		Not depressed	7		0 (0.0)	0 (0.0)	7.2 (0.4)	GW 14					
							10.1 (0.3)	GW 22					
							16.8 (0.8)	GW 30					
							18.1 (1.1)	GW 40					
Deligiannidis, 2013	17	Depressed	8	28.6 (5.9)	7 (87.5)	0 (0.0)	0.30 (0.08)^d^	PP 3–9 weeks	LC-MS/MS	Plasma	EPDS/ QIDS	10	Good
		Healthy controls	9	30.7 (3.8)	0 (0.0)	0 (0.0)	0.36 (0.12)^d^						
Hellgren, 2014	96	PDS	10	31.0 (7.7)	NA	0 (0.0)	11.61 (7.11)	GW 38.2 (0.6)	HPLC/RIA	Serum	MADRS-S/ EPDS^e^	13^e^	Fair
		Not depressed	86	30.4 (3.9)	NA	0 (0.0)	16.75 (6.31)						
Deligiannidis, 2016	56	High-risk PDS	14^f^	32.7 (5.1)	6 (42.8)	NA	11.87 (6.01)^g^	GW 29–33	LC-MS/MS	Plasma	EPDS / HDRS-17	13	Fair
							12.02 (6.08)^g^	GW 30–34					
							0.25 (0.34)^g^	PP 4.0 (0.0) weeks					
		Healthy controls	42	30.9 (4.0)	0 (0.0)	NA	12.49 (5.62)^g^	GW 29–33					
							13.92 (5.70)^g^	GW 30–34					
							0.06 (0.04)^g^	PP 4.9 (2.0) weeks					
Hellgren, 2017	292	PDS	84^f^	30.1 (4.7)	NA	21 (25.0)	6.86 (1.06)^h^	GW 12–16	HPLC/RIA	Plasma	EPDS	13	Fair
							6.15 (1.93)^h^	GW 17–20					
		Not depressed	208		NA	0 (0.0)	5.52 (2.49)^h^	GW 12–16					
							5.70 (2.00)^h^	GW 17–20					
Osborne, 2017	114	PDS	49^f^	30.5 (6.2)	49 (100.0)	10 (20.4)	2.31 (1.07)^i^	GW 12–16	ELISA	Plasma	EPDS/ MADRS	13	Fair
							2.16^i^	GW 17–20					
							2.91 (0.99)^i^	GW 21–24					
							4.23 (0.90)^i^	GW 25–28					
							5.20 (2.13)^i^	GW 29–33					
							6.50 (2.67)^i^	GW ≥ 34					
		Not depressed	65		65 (100.0)	27 (41.5)	2.78 (0.38)^i^	GW 12–16					
							3.64 (1.37)^i^	GW 17–20					
							3.15 (1.22)^i^	GW 21–24					
							4.00 (1.82)^i^	GW 25–28					
							6.40 (3.24)^i^	GW 29–33					
							8.59 (6.12)^i^	GW ≥ 34					
Guintivano, 2018	1517	PDS	549	26.6 (5.2)	292 (53.2)	NA	5.13 (5.51)^j^	PP 6 ± 1–2 weeks	ELISA	Serum	EPDS	11	Fair
		Not depressed	968	26.0 (5.9)	143 (14.8)	NA	5.03 (4.75)^j^						
Deligiannidis, 2020	88	PDS/High-risk PDS	27^f^	29.1 (4.8)	22 (81.4)	NA	7.69 (1.69)^k^	GW 25–28	LC-MS/MS	Plasma	EPDS/ HDRS-17	13	Fair
							8.93 (2.31)^k^	GW 29–33					
							10.88 (1.31)^k^	GW ≥ 34					
							1.96 (0.93)^k^	PP 1.3 (0.5) days					
							0.18 (0.19)^k^	PP 4.9 (2.5) weeks					
		Healthy controls	61	29.3 (4.9)	0 (0.0)	0 (0.0)	6.18 (1.46)^k^	GW 25–28					
							6.56 (2.67)^k^	GW 29–33					
							9.67 (2.27)^k^	GW ≥ 34					
							2.27 (1.37)^k^	PP 1.3 (0.9) days					
							0.17 (0.77)^k^	PP 5.9 (1.8) weeks					
Wenzel, 2021	50	Depressed	18^f^	29.0 (5.6)	NA	0 (0.0)	4.82 (3.37)^l^	GW 12–16	GC/MS	Serum	CAT-MH	–	Fair
							8.69^l^	GW 20–24					
							23.86 (34.06)^l^	GW 25–28					
							19.72 (13.33)^l^	GW 29–33					
		Healthy controls	32				6.43 (5.68)^l^	GW 12–16					
							6.93 (2.51)^l^	GW 20–24					
							9.10 (4.39)^l^	GW 25–28					
							10.92 (5.26)^l^	GW 29–33					
Standeven, 2022	124	PDS	31^f^	32.6 (3.7)	14 (45.2)	17 (54.8)	4.87 (2.17)^m^	GW 20–24	ELISA	Plasma	EPDS	13	Fair
							4.94 (2.48)^m^	GW 25–28					
							5.50 (1.55)^m^	GW 29–33					
							11.03 (3.33)^m^	GW ≥ 34					
							1.56 (0.58)^m^	PP 6.3 (0.7) weeks					
		Not depressed	93		10 (10.7)	4 (4.3)	7.06 (4.40)^m^	GW 20–24					
							5.37 (1.44)^m^	GW 25–28					
							9.10 (5.59)^m^	GW 29–33					
							9.88 (6.26)^m^	GW ≥ 34					
							0.80 (0.71)^m^	PP 6.0 (0.7) weeks					
Total	2509	PDS/High-risk PDS	849	27.8 (5.8)	Diagnoses: 1 No diagnoses: 3 Mixed cohorts: 9	No AD: 6 Mixed cohorts: 7		GW 12–16: 4 GW 17–20: 2 GW 21–24: 4 GW 25–28: 5 GW 29–33: 6 GW ≥ 34: 7 PP ≤ 1 week: 3 PP ≥ 2 weeks: 7	ELISA: 3 GC/MS: 2 LC-MS/MS: 3 HPLC/RIA: 3 RIA: 2	Plasma: 8 Serum: 5	CAT-MH: 1 DSM-IV:1 EPDS: 7 HDRS: 5 MADRS-S: 2 SCL-90: 1		Fair: 10 Good: 1 Poor: 2
		No PDS/healthy controls	1660										
		No peripartum healthy controls	10	28.7 (3.8)									

*AD* antidepressant, *CAT-MH* Computerized adaptive test of mental health, *ELISA* Enzyme-linked immunosorbent assay, *EPDS* Edinburgh Postnatal Depression Scale, *GC/MS* gas chromatography/mass spectrometry, *HDRS* Hamilton Depression Rating Scale, *HPLC* high performance liquid chromatography, *LC-MS/MS* Liquid Chromatography with tandem mass spectrometry, *MADRS* Montgomery-Asberg Depression Rating Scale, *MDD* major depressive disorders, *n* number of patients, *NA* not available, *PP* postpartum, *PDS* Perinatal depressive symptoms, *QIDS* Quick Inventory of Depressive Symptomatology, *RIA* radioimmunoassay, *SCL-90* Symptom Checklist-90, *SD* standard deviation, *SQ* Stein Questionnaire.

^a^Cut-off used to classify women as depressed. For two cohorts the cut-off originally used was 10 (Deligiannidis et al., 2016; Deligiannidis et al., 2020), but we reclassified patients using a cut-off of 13 (which is more broadly accepted) to increase homogeneity, as we had access to individual patient data.

^b^Peripartum assessments were drawn from a study sample of 155 patients.

^c^Data available for 7 and 12 women with vs. without depressive symptoms.

^d^Data available for 5 and 7 women with vs. without depressive symptoms.

^e^The cut-off value refers to MADRS, whereas EPDS was only delivered at postpartum.

^f^At least one time point during study period.

^g^Data available for 7 and 32, 8 and 46 as well as 2 and 44 women with vs. without depressive symptoms at 29–33 weeks, at 30–34 weeks during pregnancy and at postpartum respectively.

^f^Data available for 3 and 17, and 46 and 238 women with vs. without depressive symptoms at 12–16 weeks and at 17–20 weeks during pregnancy.

^i^Data available for 7 and 5, 1 and 6, 4 and 11, 5 and 3, 9 and 12 as well as 8 and 21 women with vs. without depressive symptoms at 12–16 weeks, 17–20 weeks, 21–24 weeks, 25–28 weeks, 29–33 weeks and 34–38 weeks respectively.

^j^Data available for 255 and 259 women with vs. without depressive symptoms.

^k^Data available for 2 and 19, 17 and 35, 12 and 58, 9 and 52 as well as 13 and 65 women with vs. without depressive symptoms at 25–28 weeks, 29–33 weeks, 34–38 weeks antepartum, at ≤1 week PP and at ≥2 weeks PP respectively.

^l^Data available for 3 and 16, 1 and 5, 5 and 27 as well as 3 and 8 women with vs. without depressive symptoms at 12–16 weeks, 20–24 weeks, 25–28 weeks and 29–33 weeks respectively.

^m^Data available for 39 and 13, 34 and 12, 3 and 35, 4 and 15 as well as 4 and 58 women with vs. without depressive symptoms at 20–24 weeks, 25–28 weeks, 29–33 weeks, 34–38 antepartum and at ≥2 weeks PP respectively.

### Primary outcome

**Fig. 1:**
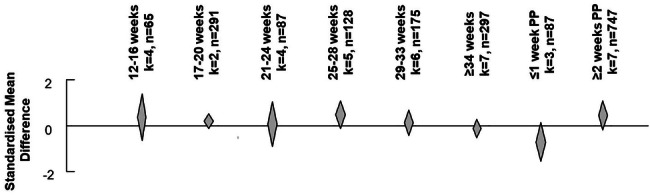
Standardized mean differences for allopregnanolone blood concentrations between women with versus without depressive symptoms at six timepoints during pregnancy and two timepoints at postpartum in random effects models. Legends: *k* number of cohorts; *n* number of women, PP postpartum.

Comparisons at <12 gestational weeks were not performed due to lack of studies. We did not detect differences for allopregnanolone concentrations between women with versus without peripartum depressive symptoms at gestational weeks 12–16 (SMD = 0.37, 95% CI = −0.65, 1.39, k = 4, *n* = 65, Fig. 1, Supplementary Fig. 2), 17–20 (SMD = 0.20, 95% CI = −0.11, 0.52, k = 2, *n* = 291, Fig. 1, Supplementary Fig. 3), 21–24 (SMD = 0.07, 95% CI = −0.90, 1.05, k = 4, *n* = 87, Fig. 1, Supplementary Fig. 4), 25–28 (SMD = 0.49, 95% CI = −0.10, 1.08, k = 5, *n* = 128, Fig. 1, Supplementary Fig. 5), 29–33 (SMD = 0.14, 95% CI = −0.41, 0.70, k = 6, *n* = 175, Fig. 1, Supplementary Fig. 6), ≥34 weeks (SMD = −0.13, 95% CI = −0.54, 0.28, k = 7, *n* = 297, Fig. 1, Supplementary Fig. 7), at postpartum ≤1 week (SMD = −0.71, 95% CI = −1.55, 0.13, k = 3, *n* = 117, Fig. 1, Supplementary Fig. 8) and ≥2 weeks postpartum (SMD = 0.46, 95% CI = −0.16, 1.07, k = 7, *n* = 747, Fig. 1, Supplementary Fig. 9). Heterogeneity was low to moderate in all cases with *I*^2^ ranging between 0 and 71%.

#### Allopregnanolone concentrations during pregnancy and depressive symptoms at postpartum follow-up

**Fig. 2:**
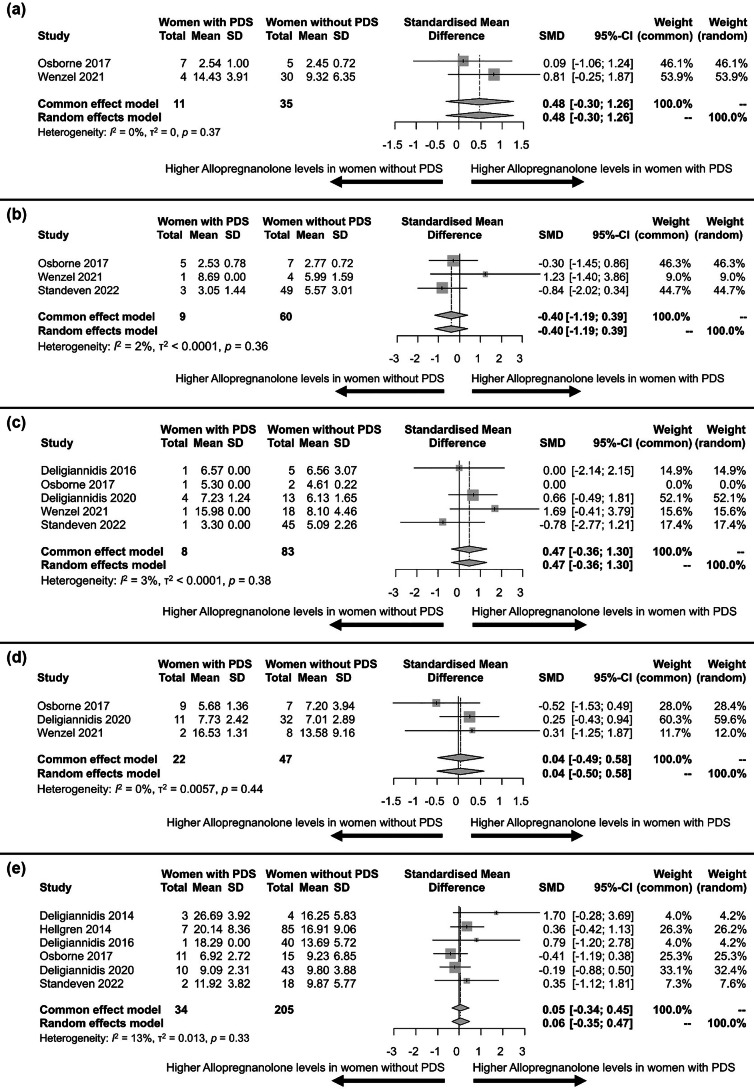
Women with vs. without depressive symptoms (PDS) at postpartum with assessed allopregnanolone blood concentrations (ng/mL). **a** At gestational weeks 12–16, **b** at gestational weeks 21–24, **c** at gestational weeks 25–28, **d** at gestational weeks 29–33, **e** at gestational weeks ≥34. CI confidence interval, SD standard deviation, SMD standardized mean difference.

We did not detect differences for allopregnanolone concentrations measured at gestational weeks 12–16, 21–24, 25–28, 29–33 or ≥34 for women with versus without depressive symptoms at postpartum ≥2 weeks postpartum with SMDs ranging between –0.40 and 0.48 (Fig. 2a–e) with low heterogeneity (*I*^2^ ranging between 0 and 13%). Comparisons were not possible for allopregnanolone assessments at gestational weeks 17–20 as there were not enough cohorts.

### Meta-regression analyses

We observed effects for maternal age, i.e. larger allopregnanolone concentration differences in samples of younger ages, at gestational weeks 29–33 (estimated co-efficient −0.42, 95% CI = −0.72, −0.11, *p* = 0.008), but not at gestational weeks 12–16 (estimated co-efficient −0.08, 95% CI = −0.64, 0.47, *p* = 0.77), at gestational weeks 21–24 (estimated co-efficient −0.14, 95% CI = −0.72, 0.43, *p* = 0.62), at gestational weeks 25–28 (estimated co-efficient −0.22, 95% CI = −0.54, 0.10, *p* = 0.18), at ≥34 gestational weeks (estimated co-efficient −0.09, 95% CI = −0.39, 0.20, *p* = 0.53), at postpartum ≤1 week (estimated co-efficient 0.07, 95% CI = −0.67, 0.78, *p* = 0.88) or ≥2 weeks (estimated co-efficient 0.19, 95% CI = −0.08, 0.47, *p* = 0.16), whereas data were not sufficiently available at gestational weeks 17–20.

### Subgroup analyses

#### Allopregnanolone concentrations in studies including women with versus without clinical diagnoses of affective disorders before pregnancy

We did not include studies with mixed cohorts, i.e. including patients with and without clinical diagnoses of affective disorders before pregnancy. Only at ≥34 gestational weeks there were more than two studies with the comparison revealing not significant differences between one study including patients with versus two studies including patients without clinical diagnoses of affective disorders before pregnancy (Table 2).

**Table 2:** Subgroup analyses.

Timepoint	GW 12–16		*I*^*2*^, %	*p*-value	GW 20–24				GW 25–28				GW 29–33				GW ≥ 34				PP ≥ 2 weeks			
	k (*n*)	SMD (95% CI)			k (*n*)	SMD (95% CI)	*I*^*2*^, %	*p*-value	k (*n*)	SMD (95% CI)	*I*^*2*^, %	*p*-value	k (*n*)	SMD (95% CI)	*I*^*2*^, %	*p*-value	k (*n*)	SMD (95% CI)	*I*^*2*^, %	*p*-value	k (*n*)	SMD (95% CI)	*I*^*2*^, %	*p*-value
Studies with patients without clinical diagnoses of affective disorders before pregnancy Studies with patients with clinical diagnoses of affective disorders before pregnancy	NP				NP				NP				NP				2 (29) 1 (29)	−0.02 (−0.75, 0.72) −0.37 (−1.19, 0.45)	0 NA	0.53	NP			
Studies using immunoassays	3 (46)	0.60 (−0.72, 1.92)	70	0.34	3 (81)	0.02 (−1.13, 1.16)	76	0.67	2 (54)	−0.13 (−0.73, 0.47)	0	**0.01**	3 (73)	−0.34 (−0.92, 0.25)	0		4 (158)	−0.38 (−0.84, 0.07)	1		2 (576)	0.42 (−0.58, 1.41)	74	
Studies using C/MS	1 (19)	−0.28 (−1.52, 0.96)	NA		1 (6)	0.56 (−1.65, 2.77)	NA		3 (74)	0.93 (0.32, 1.55)	0		3 (102)	0.57 (−0.18, 1.31)	53	0.06	3 (139)	0.11 (−0.48, 0.70)	39	0.19	5 (171)	0.53 (−0.45, 1.50)	71	0.88
Studies using plasma samples	2 (32)	−0.00 (−1.02, 1.02)	30	0.52	2 (67)	−0.62 (−1.18, −0.06)	0	**0.005**	4 (96)	0.32 (−0.31, 0.94)	24	0.19	4 (150)	0.03 (−0.70, 0.75)	69		5 (187)	0.03 (−0.40, 0.46)	12		6 (233)	0.60 (−0.19, 1.39)	68	
Studies using serum samples	2 (33)	0.76 (−1.31, 2.84)	81		2 (20)	1.07 (0.04, 2.11)	0		1 (32)	1.10 (0.10, 2.10)	NA		2 (25)	0.40 (−0.59, 1.38)	23	0.55	2 (110)	−0.51 (−1.26, 0.25)	37	0.23	1 (514)	0.02 (−0.15, 0.19)	NA	0.16

Immunoassays include enzyme-linked immunosorbent assays and radioimmunoassays; *p*-values refer to differences between groups.

*C/MS* chromatography/mass-spectrometry, *GW* gestational week, *k* number of cohorts, *n* number of women, *NA* not applicable, *NP* not performed due to lack of >2 studies, *PP* postpartum, *SMD* standardized mean difference.

Bold values mean statistical significance as they are below 0.05 (*p* < 0.05).

#### Allopregnanolone concentrations in studies including women with versus without antidepressant treatment

As there were no studies exclusively assessing antidepressant-treated patients, we were not able to perform subgroup analyses (comparing to studies including patients without antidepressant treatment). Instead, we performed a sensitivity analysis including only studies of cohorts including women without antidepressant treatment. Allopregnanolone concentrations were higher in women with versus without depressive symptoms at gestational weeks 21–24 (SMD = 1.07, 95% CI = 0.04, 2.11, k = 2, *n* = 20, *p* = 0.04) and 25–28 (SMD = 0.92, 95% CI = 0.26, 1.59, k = 2, *n* = 53, *p* = 0.007). We did not detect differences for allopregnanolone concentrations between women with versus without peripartum depressive symptoms at gestational weeks 12–16 (SMD = 0.76, 95% CI = −1.31, 2.84, k = 2, *n* = 33), 29–33 (SMD = 0.40, 95% CI = −0.59, 1.38, k = 2, *n* = 25), ≥34 weeks (SMD = −0.40, 95% CI = −0.99, 0.19, k = 3, *n* = 125), at postpartum ≤1 week (SMD = −0.96, 95% CI = −2.11, 0.19, k = 2, *n* = 56) and ≥2 weeks postpartum (SMD = −0.00, 95% CI = −0.96, 0.96, k = 2, *n* = 28). Comparisons at 17–20 gestational weeks were not performed due to lack of studies. Heterogeneity was low to moderate in all cases with I^2^ ranging between 0 and 70%, except for comparisons at 12–16 gestational weeks where I^2^ was 81%.

#### Allopregnanolone concentrations in studies using immunoassays versus chromatography/mass spectrometry methods

At gestational weeks 25–28 three studies using chromatography/mass spectrometry reported higher allopregnanolone concentrations in women with versus without depressive symptoms (SMD = 0.93, 95% CI = 0.32, 1.55, *n* = 74), whereas two studies using immunoassays reported no differences (SMD = −0.13, 95% CI = −0.73, 0.47, *n* = 54, between groups *p* = 0.01, Table 2). No differences were reported between studies using chromatography/mass spectrometry versus immunoassays at any other assessment timepoints (Table 2).

#### Allopregnanolone blood concentrations in studies using serum versus plasma samples

At gestational weeks 21–24 two studies using plasma samples reported lower allopregnanolone concentrations in women with versus without depressive symptoms (SMD = −0.62, 95% CI = −1.18, −0.06, *n* = 67), whereas two studies using serum samples reported higher allopregnanolone concentrations in women with versus without depressive symptoms (SMD = 1.07, 95% CI = 0.04, 2.11, *n* = 20, between groups *p* = 0.005, Table 2). No differences were reported between studies using chromatography/mass spectrometry versus immunoassays at any other assessment timepoints (Table 2).

### Sensitivity analyses

One study assessing women during the first week at postpartum was assessed as of poor quality [30]. As there were only two studies investigating the association between allopregnanolone concentrations and depressive symptoms at postpartum ≤1 week we did not repeat the analysis excluding this study. One additional study assessing women at gestational weeks 14, 22, 30 and 40 [53]. After excluding this study, results did not change except for the analysis at gestational weeks 21–24, where allopregnanolone concentrations were lower in women with versus without depressive symptoms (SMD = −0.55, 95% CI = −1.09, −0.00, k = 3, *n* = 73, *p* = 0.049).

### Publication bias

We did not assess the publication bias with funnel plots and Egger’s test, as we retrieved fewer than ten studies for each analysis.

## Discussion

As interest in the therapeutic potential of neuroactive steroids in treatment of postpartum depression and other neuropsychiatric disorders intensifies, the search for specific imbalances in steroid metabolism in women across the peripartum period is ongoing [35]. To our knowledge, this is the first meta-analysis to assess allopregnanolone blood concentrations in women with versus without PDS. Analyzing allopregnanolone concentration data in a cross-sectional fashion did not suggest significantly lower allopregnanolone concentrations in women with versus without peripartum depressive symptoms at any timepoint before and after delivery in alignment with some of the previous findings [32, 33, 37, 54].

Data from previous studies provided conflicting results regarding depressive symptoms and allopregnanolone concentrations late in the 2^nd^ trimester; while two studies reported lower concentrations in women with depressive symptoms late in the 2^nd^ trimester [55, 56], a third study suggested higher concentrations linked with depressive symptoms [57]. A factor accounting for this inconsistency may be the heterogeneity of the study groups; for example, one of the study samples exclusively included women with a history of affective disorders [55], whereas the other two studies included healthy controls as well [29, 56, 57]. Besides, there are essential differences with regard to the sociodemographic characteristics of the study groups, who may have different stressors influencing neuroactive steroids.

Further, we did not detect any differences for allopregnanolone concentrations during pregnancy for women with versus without longitudinally assessed depressive symptoms at postpartum. Thus, the potential of allopregnanolone concentrations (and progesterone metabolites in general) as a biomarker candidate for depressive symptoms requires further exploration. Differences for allopregnanolone concentrations between pregnant women with versus without depressive symptoms early in the 3^rd^ trimester were smaller in older women, although modifying effects for age were not reported in the rest of timepoints.

Despite the lack of differences in our main analysis, there were several striking findings in our subgroup and sensitivity analyses. For instance, we reported higher allopregnanolone concentrations in women with vs. without depressive symptoms at gestational weeks 21–24 and 25–28 when only including studies with patients not receiving antidepressant treatment. This distinct pattern may highlight the potentially modifying impact of pharmacotherapy on allopregnanolone-modulating functioning, which been understudied so far. Moreover, at gestational weeks 25–28 studies using chromatography/mass spectrometry reported higher allopregnanolone concentrations in women with versus without depressive symptoms, whereas immunoassays did not. Thus, the choice of the analytical method should not be underestimated. We specifically call for the standardization of neuroactive steroid analytic methods for two main reasons. First, there is consensus that ligand-binding assays are not available for all steroids and when available, the antigen-antibody reaction presents a possible crossover between similar molecules, which can lead to reduced specificity and interassay reproducibility [58]. For example, endogenous allopregnanolone isomers may differ in their effects on the GABA-A-R and other functions [59]. Second, ligand-binding assays measure one steroid and thus cannot provide information on related steroid metabolites. Previously, the Endocrine Society commissioned two Position Papers to highlight limitations of immunoassays and support efforts for improvement [60, 61]. We believe neuroactive steroid research should, in most cases, move away from single steroid analysis to the analysis of numerous steroids and metabolites (steroidome) using mass spectrometry-based techniques. The main advantages of mass spectrometry techniques include measurement of a large panel of steroids and pathway mapping, separation of isomers, and the use of internal standards, which ensure accuracy, precision and reproducibility.

Apart from the analytical method, it is also the choice of the types of samples, which may impact results; at gestational weeks 20–24 studies using plasma versus serum samples reported opposite patterns for allopregnanolone concentrations in women with versus without depressive symptoms. Specifically, studies using plasma samples reported lower in women with depressive symptoms, whereas higher allopregnanolone concentrations in women with versus without depressive symptoms were reported in studies using serum samples. We are not aware of any studies that previously measured allopregnanolone or other neuroactive steroid peripheral concentrations in both plasma and serum to examine the effects of sample material on measurement [62]. Progesterone (and potentially progesterone metabolites) may be sensitive to pre-analytic variables as a previous study suggested higher serum progesterone concentrations compared to plasma [50]. Additional methodological quality aspects may have confounded with the results; e.g. when repeating the analysis after excluding studies of poor quality, at gestational weeks 21–24, allopregnanolone concentrations were lower in women with versus without depressive symptoms.

There are several limitations to our findings. As already highlighted, heterogeneity of participants (including variation in mood disorder history and medication use) may have affected results. Studies addressing new onset peripartum depressive symptoms may provide differentiated knowledge after eliminating the confounder of history of affective symptoms. Another potential confounder that should be investigated in the future refers to the role of common obstetric complications. As some obstetric complications are risk factors for peripartum depressive symptoms [63], it is substantially important to disentangle their impact on the interplay between peripartum depressive symptoms and allopregnanolone concentrations [64]. While some of the studies included here did consider this factor, effects were not discernible given the small numbers of women having such complications. Moreover, information on pregnancy outcomes was lacking in the majority of the studies and thus we were unable to consider their role. Given the small number of studies we could not assess (and thus rule out) the risk of publication bias; however, as our search of published and unpublished studies was systematic and it is less common to find publication bias in case of systematic reviews of biological blood measurement studies, there is no suspicion of publication bias.

As alluded to earlier, study cohorts were heterogeneous, and some were less than optimally characterized. Regarding heterogeneity of the recruited cohorts, research to date has included women with a history of affective/anxiety symptoms who were at-risk for PDS, women with current or past unipolar depression and bipolar disorder, women with current PDS who were or were not receiving psychopharmacological treatments (including some known to affect neurosteroidogenesis). Longitudinal studies across gestation, with some continued sampling into the postpartum period, tended to be of a small sample size, with the largest PDS sample size of 84 [54]. The largest PDS sample was from a cross-sectional postpartum study with a PDS sample size of 549 [37]. However, often power analyses were not included in the publications, resulting is poorer quality rating. Only with larger, higher-quality, well-phenotyped studies will we unravel the potential differences in peripartum allopregnanolone concentrations, with an ultimate aim to fully characterize steroid metabolism profiles, amongst women with phenomenologically distinct subtypes of peripartum depression [65]. We recommend that future research should assess for potential modifiers of neurosteroidogenesis including a history of trauma, alcohol use disorder, or major depression, each of which are risk factors for peripartum depression [38, 66, 67]. Additionally, the cohorts included in this analysis varied considerably in terms of timing of PDS onset. PDS or peripartum depression with antepartum vs. postpartum onset could be biologically quite different phenotypes, and may warrant separate analysis, given data that suggests the existence of different types and severity of peripartum depression with varying time of onset throughout pregnancy and the postpartum [65, 68]. Sample timing is also important given the diurnal variation of allopregnanolone [69]. Finally, some studies distinguished between concurrent and future symptoms, whereas other studies included in their groups with depressive symptoms those who developed symptoms at any timepoint.

To improve study rigor and quality, we recommend that future studies should exclude from analysis, or analyze separately (depending upon the research question), participants taking pharmacotherapies or with medical conditions known to affect either brain neurosteroidogenesis and/or impact peripheral blood concentrations. This should include not only psychotropics but other commonly prescribed medications in the peripartum period including progesterone supplementation to reduce the risk of spontaneous preterm labor and birth and antenatal corticosteroid therapy administered to patients at risk for preterm labor and birth to reduce the incidence and severity of respiratory distress syndrome and offspring mortality. Similarly, neurological, endocrine and other medical conditions (such as polycystic ovary syndrome and inflammatory conditions), which may impact neurosteroidogenesis and/or impact peripheral blood concentrations [70, 71] should be measured and/or controlled for.

Additionally, to improve sample phenotyping, we recommend studies to include further measures, e.g., observer-rating scales derived from validated semi-structured interviews, complemented by psychiatric diagnostic interviews or behavioral measures. Most studies in our analysis utilized the EPDS which is commonly used in clinical settings to screen for peripartum depression. The use of the EPDS in the second trimester of pregnancy identifies a significant number of women with psychiatric disorders other than depression, such as bipolar disorder, obsessive-compulsive disorder and eating disorders, making it important to additionally have diagnostic data that complements self-report and screening measures [72]. Given the heterogeneity, and comorbidity, of most psychiatric disorders, including peripartum depression, it will be important to measure and integrate multiple dimensions (e.g., cognition, mood, mother-infant bonding) and units of analysis across a range of severity to explore dimensions of functioning that occur within or across current diagnostic boundaries. Diagnostic data can aid in our interpretation of the neuroactive steroid data, as the presence of comorbid psychiatric diagnoses might reflect different patterns of symptoms that result from shared risk factors and perhaps shared steroid metabolic profiles.

The strength of our meta-analysis is the inclusion of multiple timepoints accounting for the dynamic nature of neuroactive steroids and the GABAergic system. In summary, our meta-analytical evidence suggested lower allopregnanolone concentrations in women with depressive symptoms compared to women without at gestational weeks 21–24. At all other timepoints we did not identify any distinct patterns for allopregnanolone blood concentrations in women with peripartum depressive symptoms compared to women without. Advancing knowledge of factors that underpin potential peripheral and central deficits of progesterone derivates, such as allopregnanolone, is important to determine the neurobiological mechanisms of peripartum depressive symptoms and ultimately to contribute to improvement of early identification, management, and treatment for women suffering the most common psychiatric complication of childbirth.

## Supplementary information

Supplementary Material

MOOSE Checklist

## References

[1] Trost S, Beauregard J, Chandra G, Njie F, Berry J, Harvey A, et al. Pregnancy-Related Deaths: Data from Maternal Mortality Review Committees in 36 US States, 2017–2019 Atlanta, GA: Centers for Disease Control and Prevention UDoHaHS; 2022. Available from: https://www.cdc.gov/reproductivehealth/maternal-mortality/erase-mm/data-mmrc.html.

[2] Knight M, Bunch K, Tuffnell D, Patel R, Shakespeare J, Kotnis R, et al. Saving Lives, Improving Mothers’ Care - Lessons learned to inform maternity care from the UK and Ireland Confidential Enquiries into Maternal Deaths and Morbidity 2017-19 Oxford, UK: National Perinatal Epidemiology Unit, University of Oxford; 2021. https://www.npeu.ox.ac.uk/assets/downloads/mbrrace-uk/reports/maternal-report-2021/MBRRACE-UK_Maternal_Report_2021_-_FINAL_-_WEB_VERSION.pdf.

[3] Liu X, Wang S, Wang G. Prevalence and Risk Factors of Postpartum Depression in Women: A Systematic Review and Meta-analysis. J Clin Nurs. 2022;31:2665–77. 34750904 10.1111/jocn.16121

[4] Yonkers KA, Vigod S, Ross LE. Diagnosis, pathophysiology, and management of mood disorders in pregnant and postpartum women. Obstet Gynecol. 2011;117:961–77. 21422871 10.1097/AOG.0b013e31821187a7

[5] Field T. Postpartum depression effects on early interactions, parenting, and safety practices: a review. Infant Behav Dev. 2010;33:1–6. 19962196 10.1016/j.infbeh.2009.10.005 PMC2819576

[6] Perry DF, Ettinger AK, Mendelson T, Le HN. Prenatal depression predicts postpartum maternal attachment in low-income Latina mothers with infants. Infant Behav Dev. 2011;34:339–50. 21402409 10.1016/j.infbeh.2011.02.005

[7] Dennis CL, McQueen K. The relationship between infant-feeding outcomes and postpartum depression: a qualitative systematic review. Pediatrics. 2009;123:e736–51. 19336362 10.1542/peds.2008-1629

[8] Tuovinen S, Lahti-Pulkkinen M, Girchenko P, Lipsanen J, Lahti J, Heinonen K, et al. Maternal depressive symptoms during and after pregnancy and child developmental milestones. Depress Anxiety. 2018;35:732–41. 29667739 10.1002/da.22756

[9] Leis JA, Heron J, Stuart EA, Mendelson T. Associations between maternal mental health and child emotional and behavioral problems: does prenatal mental health matter? J Abnorm Child Psychol. 2014;42:161–71. 23748337 10.1007/s10802-013-9766-4

[10] Pearson RM, Evans J, Kounali D, Lewis G, Heron J, Ramchandani PG, et al. Maternal depression during pregnancy and the postnatal period: risks and possible mechanisms for offspring depression at age 18 years. JAMA Psychiatry. 2013;70:1312–9. 24108418 10.1001/jamapsychiatry.2013.2163 PMC3930009

[11] Schweizer-Schubert S, Gordon JL, Eisenlohr-Moul TA, Meltzer-Brody S, Schmalenberger KM, Slopien R, et al. Steroid Hormone Sensitivity in Reproductive Mood Disorders: On the Role of the GABA(A) Receptor Complex and Stress During Hormonal Transitions. Front Med. 2020;7:479646. 10.3389/fmed.2020.479646 PMC7873927 33585496

[12] Antonoudiou P, Colmers PLW, Walton NL, Weiss GL, Smith AC, Nguyen DP, et al. Allopregnanolone Mediates Affective Switching Through Modulation of Oscillatory States in the Basolateral Amygdala. Biol Psychiatry. 2022;91:283–93. 34561029 10.1016/j.biopsych.2021.07.017 PMC8714669

[13] Callachan H, Cottrell GA, Hather NY, Lambert JJ, Nooney JM, Peters JA. Modulation of the GABAA receptor by progesterone metabolites. Proc R Soc Lond B Biol Sci. 1987;231:359–69. 2888123 10.1098/rspb.1987.0049

[14] Avoli M, Krnjevic K. The Long and Winding Road to Gamma-Amino-Butyric Acid as Neurotransmitter. Can J Neurol Sci. 2016;43:219–26. 26763167 10.1017/cjn.2015.333

[15] Parakala ML, Zhang Y, Modgil A, Chadchankar J, Vien TN, Ackley MA, et al. Metabotropic, but not allosteric, effects of neurosteroids on GABAergic inhibition depend on the phosphorylation of GABA(A) receptors. J Biol Chem. 2019;294:12220–30. 31239352 10.1074/jbc.RA119.008875 PMC6690684

[16] Semyanov A, Walker MC, Kullmann DM. GABA uptake regulates cortical excitability via cell type-specific tonic inhibition. Nat Neurosci. 2003;6:484–90. 12679782 10.1038/nn1043

[17] Stell BM, Brickley SG, Tang CY, Farrant M, Mody I. Neuroactive steroids reduce neuronal excitability by selectively enhancing tonic inhibition mediated by delta subunit-containing GABAA receptors. Proc Natl Acad Sci USA. 2003;100:14439–44. 14623958 10.1073/pnas.2435457100 PMC283610

[18] Crowley SK, Girdler SS. Neurosteroid, GABAergic and hypothalamic pituitary adrenal (HPA) axis regulation: what is the current state of knowledge in humans? Psychopharmacology. 2014;231:3619–34. 24756763 10.1007/s00213-014-3572-8 PMC4135030

[19] Sarkar J, Wakefield S, MacKenzie G, Moss SJ, Maguire J. Neurosteroidogenesis is required for the physiological response to stress: role of neurosteroid-sensitive GABAA receptors. J Neurosci. 2011;31:18198–210. 22171026 10.1523/JNEUROSCI.2560-11.2011 PMC3272883

[20] Walton NL, Antonoudiou P, Maguire JL. Neurosteroid influence on affective tone. Neurosci Biobehav Rev. 2023;152:105327. 37499891 10.1016/j.neubiorev.2023.105327 PMC10528596

[21] Pisu MG, Concas L, Siddi C, Serra M, Porcu P. The Allopregnanolone Response to Acute Stress in Females: Preclinical and Clinical Studies. Biomolecules. 2022;12:1262. 10.3390/biom12091262 PMC9496329 36139100

[22] Cook JB, Dumitru AM, O’Buckley TK, Morrow AL. Ethanol administration produces divergent changes in GABAergic neuroactive steroid immunohistochemistry in the rat brain. Alcohol Clin Exp Res. 2014;38:90–9. 23906006 10.1111/acer.12223 PMC4196317

[23] Kanes S, Colquhoun H, Gunduz-Bruce H, Raines S, Arnold R, Schacterle A, et al. Brexanolone (SAGE-547 injection) in post-partum depression: a randomised controlled trial. Lancet. 2017;390:480–9. 28619476 10.1016/S0140-6736(17)31264-3

[24] Meltzer-Brody S, Colquhoun H, Riesenberg R, Epperson CN, Deligiannidis KM, Rubinow DR, et al. Brexanolone injection in post-partum depression: two multicentre, double-blind, randomised, placebo-controlled, phase 3 trials. Lancet. 2018;392:1058–70. 30177236 10.1016/S0140-6736(18)31551-4

[25] Deligiannidis KM, Citrome L, Huang MY, Acaster S, Fridman M, Bonthapally V, et al. Effect of Zuranolone on Concurrent Anxiety and Insomnia Symptoms in Women With Postpartum Depression. J Clin Psychiatry. 2023;84:22m14475. 10.4088/JCP.22m14475 36724109

[26] Epperson CN, Rubinow DR, Meltzer-Brody S, Deligiannidis KM, Riesenberg R, Krystal AD, et al. Effect of brexanolone on depressive symptoms, anxiety, and insomnia in women with postpartum depression: Pooled analyses from 3 double-blind, randomized, placebo-controlled clinical trials in the HUMMINGBIRD clinical program. J Affect Disord. 2023;320:353–9. 36191643 10.1016/j.jad.2022.09.143

[27] Deligiannidis KM, Meltzer-Brody S, Gunduz-Bruce H, Doherty J, Jonas J, Li S, et al. Effect of Zuranolone vs Placebo in Postpartum Depression: A Randomized Clinical Trial. JAMA Psychiatry. 2021;78:951–9. 34190962 10.1001/jamapsychiatry.2021.1559 PMC8246337

[28] Deligiannidis KM, Meltzer-Brody S, Maximos B, Peeper EQ, Freeman M, Lasser R, et al. Zuranolone for the Treatment of Postpartum Depression. Am J Psychiatry. 2023;180:668–75. 37491938 10.1176/appi.ajp.20220785

[29] Osborne LM, Betz JF, Yenokyan G, Standeven LR, Payne JL. The Role of Allopregnanolone in Pregnancy in Predicting Postpartum Anxiety Symptoms. Front Psychol. 2019;10:1033. 31379633 10.3389/fpsyg.2019.01033 PMC6646409

[30] Nappi RE, Petraglia F, Luisi S, Polatti F, Farina C, Genazzani AR. Serum allopregnanolone in women with postpartum “blues”. Obstet Gynecol. 2001;97:77–80. 11152912 10.1016/s0029-7844(00)01112-1

[31] Hellgren C, Akerud H, Skalkidou A, Backstrom T, Sundstrom-Poromaa I. Low serum allopregnanolone is associated with symptoms of depression in late pregnancy. Neuropsychobiology. 2014;69:147–53. 24776841 10.1159/000358838

[32] Epperson CN, Gueorguieva R, Czarkowski KA, Stiklus S, Sellers E, Krystal JH, et al. Preliminary evidence of reduced occipital GABA concentrations in puerperal women: a 1H-MRS study. Psychopharmacology. 2006;186:425–33. 16724188 10.1007/s00213-006-0313-7

[33] Deligiannidis KM, Sikoglu EM, Shaffer SA, Frederick B, Svenson AE, Kopoyan A, et al. GABAergic neuroactive steroids and resting-state functional connectivity in postpartum depression: a preliminary study. J Psychiatr Res. 2013;47:816–28. 23499388 10.1016/j.jpsychires.2013.02.010 PMC3983790

[34] Maguire J, Mody I. GABA(A)R plasticity during pregnancy: relevance to postpartum depression. Neuron. 2008;59:207–13. 18667149 10.1016/j.neuron.2008.06.019 PMC2875248

[35] Walton NL, Antonoudiou P, Barros L, Dargan T, DiLeo A, Evans-Strong A, et al. Impaired Endogenous Neurosteroid Signaling Contributes to Behavioral Deficits Associated With Chronic Stress. Biol Psychiatry. 2023;94:249–61. 10.1016/j.biopsych.2023.01.022 PMC10363189 36736870

[36] Deligiannidis KM, Kroll-Desrosiers AR, Mo S, Nguyen HP, Svenson A, Jaitly N, et al. Peripartum neuroactive steroid and gamma-aminobutyric acid profiles in women at-risk for postpartum depression. Psychoneuroendocrinology. 2016;70:98–107. 27209438 10.1016/j.psyneuen.2016.05.010 PMC4907817

[37] Guintivano J, Sullivan PF, Stuebe AM, Penders T, Thorp J, Rubinow DR, et al. Adverse life events, psychiatric history, and biological predictors of postpartum depression in an ethnically diverse sample of postpartum women. Psychol Med. 2018;48:1190–200. 28950923 10.1017/S0033291717002641 PMC6792292

[38] Deligiannidis KM, Kroll-Desrosiers AR, Tan Y, Dubuke ML, Shaffer SA. Longitudinal proneuroactive and neuroactive steroid profiles in medication-free women with, without and at-risk for perinatal depression: A liquid chromatography-tandem mass spectrometry analysis. Psychoneuroendocrinology. 2020;121:104827. 32828068 10.1016/j.psyneuen.2020.104827 PMC7572700

[39] Schule C, Baghai TC, di Michele F, Eser D, Pasini A, Schwarz M, et al. Effects of combination treatment with mood stabilizers and mirtazapine on plasma concentrations of neuroactive steroids in depressed patients. Psychoneuroendocrinology. 2007;32:669–80. 17560730 10.1016/j.psyneuen.2007.04.004

[40] Strohle A, Romeo E, Hermann B, Pasini A, Spalletta G, di Michele F, et al. Concentrations of 3 alpha-reduced neuroactive steroids and their precursors in plasma of patients with major depression and after clinical recovery. Biol Psychiatry. 1999;45:274–7. 10023501 10.1016/s0006-3223(98)00328-x

[41] Stroup DF, Berlin JA, Morton SC, Olkin I, Williamson GD, Rennie D, et al. Meta-analysis of observational studies in epidemiology: a proposal for reporting. Meta-analysis Of Observational Studies in Epidemiology (MOOSE) group. JAMA. 2000;283:2008–12. 10789670 10.1001/jama.283.15.2008

[42] Cox JL, Holden JM, Sagovsky R. Detection of postnatal depression. Development of the 10-item Edinburgh Postnatal Depression Scale. Br J Psychiatry. 1987;150:782–6. 3651732 10.1192/bjp.150.6.782

[43] Hill M, Bicikova M, Parizek A, Havlikova H, Klak J, Fajt T, et al. Neuroactive steroids, their precursors and polar conjugates during parturition and postpartum in maternal blood: 2. Time profiles of pregnanolone isomers. J Steroid Biochem Mol Biol. 2001;78:51–7. 11530284 10.1016/s0960-0760(01)00073-5

[44] Parizek A, Hill M, Kancheva R, Havlikova H, Kancheva L, Cindr J, et al. Neuroactive pregnanolone isomers during pregnancy. J Clin Endocrinol Metab. 2005;90:395–403. 15486056 10.1210/jc.2004-0444

[45] Herzog R, Alvarez-Pasquin MJ, Diaz C, Del Barrio JL, Estrada JM, Gil A. Are healthcare workers’ intentions to vaccinate related to their knowledge, beliefs and attitudes? A systematic review. BMC Public Health. 2013;13:154. 23421987 10.1186/1471-2458-13-154 PMC3602084

[46] Schoretsanitis G, Nikolakopoulou A, Guinart D, Correll CU, Kane JM. Iron homeostasis alterations and risk for akathisia in patients treated with antipsychotics: A systematic review and meta-analysis of cross-sectional studies. Eur Neuropsychopharmacol. 2020;35:1–11. 32444336 10.1016/j.euroneuro.2020.04.001

[47] DerSimonian R, Laird N. Meta-analysis in clinical trials. Control Clin Trials. 1986;7:177–88. 3802833 10.1016/0197-2456(86)90046-2

[48] Higgins JP, Thompson SG, Deeks JJ, Altman DG. Measuring inconsistency in meta-analyses. BMJ. 2003;327:557–60. 12958120 10.1136/bmj.327.7414.557 PMC192859

[49] Borenstein M, Hedges LV, Higgins JP, Rothstein HR. Meta-Regression. In: Borenstein M, Hedges LV, Higgins JP, Rothstein HR, editors. Introduction to Meta‐Analysis: New Jersey, US: John Wiley & Sons; 2009.

[50] Thuroczy J, Wolfling A, Tibold A, Balogh L, Janoki GA, Solti L. Effect of anticoagulants and sampling time on results of progesterone determination in canine blood samples. Reprod Domest Anim. 2003;38:386–9. 12950690 10.1046/j.1439-0531.2003.00450.x

[51] Egger M, Davey Smith G, Schneider M, Minder C. Bias in meta-analysis detected by a simple, graphical test. BMJ. 1997;315:629–34. 9310563 10.1136/bmj.315.7109.629 PMC2127453

[52] Schwarzer G, Carpenter JR, Rücker G. Meta-Analysis with R. Heidelberg: Springer; 2015.

[53] Paoletti AM, Romagnino S, Contu R, Orru MM, Marotto MF, Zedda P, et al. Observational study on the stability of the psychological status during normal pregnancy and increased blood levels of neuroactive steroids with GABA-A receptor agonist activity. Psychoneuroendocrinology. 2006;31:485–92. 16406349 10.1016/j.psyneuen.2005.11.006

[54] Hellgren C, Comasco E, Skalkidou A, Sundstrom-Poromaa I. Allopregnanolone levels and depressive symptoms during pregnancy in relation to single nucleotide polymorphisms in the allopregnanolone synthesis pathway. Horm Behav. 2017;94:106–13. 28666923 10.1016/j.yhbeh.2017.06.008

[55] Osborne LM, Gispen F, Sanyal A, Yenokyan G, Meilman S, Payne JL. Lower allopregnanolone during pregnancy predicts postpartum depression: An exploratory study. Psychoneuroendocrinology. 2017;79:116–21. 28278440 10.1016/j.psyneuen.2017.02.012 PMC5420429

[56] Standeven LR, Osborne LM, Betz JF, Yenokyan G, Voegtline K, Hantsoo L, et al. Allopregnanolone and depression and anxiety symptoms across the peripartum: an exploratory study. Arch Womens Ment Health. 2022;25:521–6. 10.1007/s00737-021-01186-5 PMC9113043 34714413

[57] Wenzel ES, Pinna G, Eisenlohr-Moul T, Bernabe BP, Tallon RR, Nagelli U, et al. Neuroactive steroids and depression in early pregnancy. Psychoneuroendocrinology. 2021;134:105424. 34607173 10.1016/j.psyneuen.2021.105424 PMC8943472

[58] Kaleta M, Oklestkova J, Novak O, Strnad M. Analytical Methods for the Determination of Neuroactive Steroids. Biomolecules 2021;11:553. 10.3390/biom11040553 PMC8068886 33918915

[59] Tateiwa H, Chintala SM, Chen Z, Wang L, Amtashar F, Bracamontes J, et al. The Mechanism of Enantioselective Neurosteroid Actions on GABA(A) Receptors. Biomolecules. 2023;13:341. 10.3390/biom13020341 PMC9953308 36830708

[60] Rosner W, Hankinson SE, Sluss PM, Vesper HW, Wierman ME. Challenges to the measurement of estradiol: an endocrine society position statement. J Clin Endocrinol Metab. 2013;98:1376–87. 23463657 10.1210/jc.2012-3780 PMC3615207

[61] Rosner W, Auchus RJ, Azziz R, Sluss PM, Raff H. Position statement: Utility, limitations, and pitfalls in measuring testosterone: an Endocrine Society position statement. J Clin Endocrinol Metab. 2007;92:405–13. 17090633 10.1210/jc.2006-1864

[62] Liu X, Hoene M, Wang X, Yin P, Haring HU, Xu G, et al. Serum or plasma, what is the difference? Investigations to facilitate the sample material selection decision making process for metabolomics studies and beyond. Anal Chim Acta. 2018;1037:293–300. 30292305 10.1016/j.aca.2018.03.009

[63] Gastaldon C, Solmi M, Correll CU, Barbui C, Schoretsanitis G. Risk factors of postpartum depression and depressive symptoms: umbrella review of current evidence from systematic reviews and meta-analyses of observational studies. Br J Psychiatry. 2022;221:591–602. 10.1192/bjp.2021.222 35081993

[64] Gilbert Evans SE, Ross LE, Sellers EM, Purdy RH, Romach MK. 3alpha-reduced neuroactive steroids and their precursors during pregnancy and the postpartum period. Gynecol Endocrinol. 2005;21:268–79. 16373246 10.1080/09513590500361747

[65] Putnam KT, Wilcox M, Robertson-Blackmore E, Sharkey K, Bergink V, Munk-Olsen T, et al. Clinical phenotypes of perinatal depression and time of symptom onset: analysis of data from an international consortium. Lancet Psychiatry. 2017;4:477–85. 28476427 10.1016/S2215-0366(17)30136-0 PMC5836292

[66] Pierucci-Lagha A, Covault J, Feinn R, Khisti RT, Morrow AL, Marx CE, et al. Subjective effects and changes in steroid hormone concentrations in humans following acute consumption of alcohol. Psychopharmacology. 2006;186:451–61. 16341848 10.1007/s00213-005-0231-0

[67] Pineles SL, Nillni YI, Pinna G, Webb A, Arditte Hall KA, Fonda JR, et al. Associations between PTSD-Related extinction retention deficits in women and plasma steroids that modulate brain GABA(A) and NMDA receptor activity. Neurobiol Stress. 2020;13:100225. 32490055 10.1016/j.ynstr.2020.100225 PMC7256058

[68] Postpartum Depression: Action Towards C, Treatment C. Heterogeneity of postpartum depression: a latent class analysis. Lancet Psychiatry. 2015;2:59–67. 26359613 10.1016/S2215-0366(14)00055-8 PMC4800818

[69] Segebladh B, Bannbers E, Moby L, Nyberg S, Bixo M, Backstrom T, et al. Allopregnanolone serum concentrations and diurnal cortisol secretion in women with premenstrual dysphoric disorder. Arch Womens Ment Health. 2013;16:131–7. 23329007 10.1007/s00737-013-0327-1

[70] Hedstrom H, Backstrom T, Bixo M, Nyberg S, Wang M, Gideonsson I, et al. Women with polycystic ovary syndrome have elevated serum concentrations of and altered GABA(A) receptor sensitivity to allopregnanolone. Clin Endocrinol. 2015;83:643–50. 10.1111/cen.12809 25929428

[71] Noorbakhsh F, Baker GB, Power C. Allopregnanolone and neuroinflammation: a focus on multiple sclerosis. Front Cell Neurosci. 2014;8:134. 24917787 10.3389/fncel.2014.00134 PMC4042158

[72] Lydsdottir LB, Howard LM, Olafsdottir H, Thome M, Tyrfingsson P, Sigurdsson JF. The mental health characteristics of pregnant women with depressive symptoms identified by the Edinburgh Postnatal Depression Scale. J Clin Psychiatry. 2014;75:393–8. 24569071 10.4088/JCP.13m08646

