# Supplementary material for: Peripartum allopregnanolone blood concentrations and depressive symptoms: a systematic review and individual participant data meta-analysis
Source: Mol Psychiatry. 2024 Nov 7;30(3):1148–60. doi: 10.1038/s41380-024-02747-7 (PMC11835716; doi:10.1038/s41380-024-02747-7)
Supplement: Supplementary file 1 — Supplementary Material [file 41380_2024_2747_MOESM1_ESM.docx]

**Peripartum allopregnanolone blood concentrations and depressive symptoms:**

**a systematic review and individual participant data meta-analysis**

Georgios Schoretsanitis, MD, PhD; Lauren M. Osborne, MD; Inger Sundström-Poromaa, MD; Elizabeth S. Wenzel, PhD; Jennifer Payne, MD; Corrado Barbui, MD; Chiara Gastaldon, MD, PhD; Kristina M. Deligiannidis, MD

**Supplementary Material**

**Contents**

[**List of excluded studies after full-text screening** 3](#_Toc173085138)

[**Supplementary Figure 1.** Study flow diagram 5](#_Toc173085139)

[**Supplementary Figure 2.** Allopregnanolone blood levels (ng/mL) in women with vs. without peripartum depressive symptoms (PDS) at gestational weeks 12-16. CI: confidence interval; SD: standard deviation; SMD: standardized mean difference 6](#_Toc173085140)

[**Supplementary Figure 3.** Allopregnanolone blood levels (ng/mL) in women with vs. without peripartum depressive symptoms (PDS) at gestational weeks 17-20. CI: confidence interval; SD: standard deviation; SMD: standardized mean difference 7](#_Toc173085141)

[**Supplementary Figure 4.** Allopregnanolone blood levels (ng/mL) in women with vs. without peripartum depressive symptoms (PDS) at gestational weeks 21-24. CI: confidence interval; SD: standard deviation; SMD: standardized mean difference 8](#_Toc173085142)

[**Supplementary Figure 5.** Allopregnanolone blood levels (ng/mL) in women with vs. without peripartum depressive symptoms (PDS) at gestational weeks 25-28. CI: confidence interval; SD: standard deviation; SMD: standardized mean difference 9](#_Toc173085143)

[**Supplementary Figure 6.** Allopregnanolone blood levels (ng/mL) in women with vs. without peripartum depressive symptoms (PDS) at gestational weeks 29-33. CI: confidence interval; SD: standard deviation; SMD: standardized mean difference 10](#_Toc173085144)

[**Supplementary Figure 7.** Allopregnanolone blood levels (ng/mL) in women with vs. without peripartum depressive symptoms (PDS) at ≥34 gestational weeks. CI: confidence interval; SD: standard deviation; SMD: standardized mean difference 11](#_Toc173085145)

[**Supplementary Figure 8.** Allopregnanolone blood levels (ng/mL) in women with vs. without peripartum depressive symptoms (PDS) at ≤1 week postpartum. CI: confidence interval; SD: standard deviation; SMD: standardized mean difference 12](#_Toc173085146)

[**Supplementary Figure 9.** Allopregnanolone blood levels (ng/mL) in women with vs. without peripartum depressive symptoms (PDS) ≥2 weeks at postpartum. CI: confidence interval; SD: standard deviation; SMD: standardized mean difference 13](#_Toc173085147)

[**Supplementary Table 1.** Quality of included studies 14](#_Toc173085148)

# **List of excluded studies after full-text screening**

| **Reference** | **Reason for exclusion** |
| --- | --- |
| Gilbert Evans et al. 3alpha-reduced neuroactive steroids and their precursors during pregnancy and the postpartum period. Gynecol Endocrinol. 2005;21(5):268-79 | Allopregnanolone levels not stratified for women with vs. without PDS |
| Almeida et al. The Role of HPA Axis and Allopregnanolone on the Neurobiology of Major Depressive Disorders and PTSD. Int J Mol Sci. 2021; 22(11):5495 | Review |
| Bendix et al. Allopregnanolone and progesterone in estradiol treated severe postpartum affective disorder. Psychoneuroendocrinology. 2019;107: S68 | Effects of estradiol treatment on allopregnanolone levels |
| Bendix et al. Allopregnanolone and progesterone in estradiol treated severe postpartum depression and psychosis – Preliminary findings. Neurol Psych Brain Res. 2019;34:50-57 | Effects of estradiol treatment on allopregnanolone levels |
| Deligiannidis et al. Resting-state functional connectivity and neuroactive steroids in postpartum depression. Neuropsychopharmacology. 2012;38:S249-50 | Overlapping data |
| Deligiannidis et al. Resting-State Functional Connectivity Cortical Gaba and Allopregnanolone in Postpartum Depression: A Functional Magnetic Imaging and Spectroscopy Study. Biol Psychiatry. 2019;85(10):S114 | Overlapping data |
| Deligiannidis et al. Rapid ultrasensitive high precision LC-MS assays identify unique neuroactive steroid and GABA profiles in women at-risk for postpartum depression. Neuropsychopharmacology. 2015:;40:S153-S154 | Overlapping data |
| Deligiannidis et al. Peripartum neuroactive steroid and γ-aminobutyric acid profiles in women at-risk for postpartum depression. Psychoneuroendocrinol. 2016;70:98-107 | Overlapping data |
| Frye et al. 3alpha-hydroxy-5alpha-pregnan-20-one in the midbrain ventral tegmental area mediates social, sexual, and affective behaviors. Neuroscience. 2006;138(3):1007-14 | Review |
| Grötsch MK, Ehlert U. Allopregnanolone in the peripartum: Correlates, concentrations, and challenges - A systematic review. Psychoneuroendocrinology. 2024 Aug;166:107081. | Review |
| Hardoy et al. Increased neuroactive steroid concentrations in women with bipolar disorder or major depressive disorder. J Clin Psychopharmacol. 2006;26(4):379-84. | No peripartum assessments |
| Hellgren et al. Low serum allopregnanolone is associated with elevated depressive symptoms in late pregnancy. Eur Neuropsychopharmacol. 2012; 22(2):S228 | Overlapping data |
| Klatzkin et al. Associations of histories of depression and PMDD diagnosis with allopregnanolone concentrations following the oral administration of micronized progesterone. Psychoneuroendocrinology. 2006;31(10):1208-19 | No peripartum assessments |
| Osborne et al. Increased inflammatory markers in late pregnancy depression: The role of progesterone and allopregnanolone. Brain Behav Immun. 2015;49S:e22-3 | Overlapping data |
| Osborne et al. Inflammatory and hormonal markers of peripartum depression. Biol Psychiatry. 2016;79(9):S142 | Overlapping data |
| Osborne et al. Replication of epigenetic postpartum depression biomarkers and variation with hormone levels. Neuropsychopharmacology. 2016;41(6):1648-58 | Overlapping data |
| Osborne et al. The Role of Allopregnanolone in Perinatal Mood and Anxiety Disorders. Biol Psychiatry. 2019;85(10):S15-16 | Overlapping data |
| Osborne et al. Lower Allopregnanolone in pregnancy predicts postpartum depression. Arch Womens Ment Health. 2020;23:247 | Overlapping data |
| Payne. Evaluating brexanolone for the treatment of postpartum depression. Expert Opin Pharmacother. 2021;22(8):959-964 | Review |
| Schweizer-Schubert et al. Steroid Hormone Sensitivity in Reproductive Mood Disorders: On the Role of the GABA(A) Receptor Complex and Stress During Hormonal Transitions. Front Med. 2021;7:479646 | Review |
| Thitipitchayanant et al. Effectiveness of self-empowerment-affirmation-relaxation (Self-EAR) program for postpartum blues mothers: A randomize controlled trial. Pak J Med Sci. 2018;34(6):1488-1493 | Effects of a psychological intervention on allopregnanolone levels |
| Wirth. Beyond the HPA Axis: Progesterone-Derived Neuroactive Steroids in Human Stress and Emotion. Front Endocrinol (Lausanne). 2011;2:19 | Review |

# **Supplementary Figure 1.** Study flow diagram

Studies included in the systematic review and meta-analysis
(n = 13)

Records screened
(n = 276)

Full-text articles assessed for eligibility
(n = 35)

Articles excluded based on title and abstract

(n = 241)

Screening

Included

Eligibility

Identification

Records identified through updated database searching
(Medline: n = 3)

Records identified through database searching
(Medline: n = 203, Embase: n = 94)

Full-text articles excluded with reason (n=22): overlapping data (n = 10),

reviews (n=6), effects of interventions on allopregnanolone levels (n=3),

no perinatal assessments (n=2),

lack of stratified allopregnanolone levels for women with vs. without perinatal depressive symptoms (n=1)

Duplicates removed

(n = 24)

# **Supplementary Figure 2.** Allopregnanolone blood levels (ng/mL) in women with vs. without peripartum depressive symptoms (PDS) at gestational weeks 12-16. CI: confidence interval; SD: standard deviation; SMD: standardized mean difference


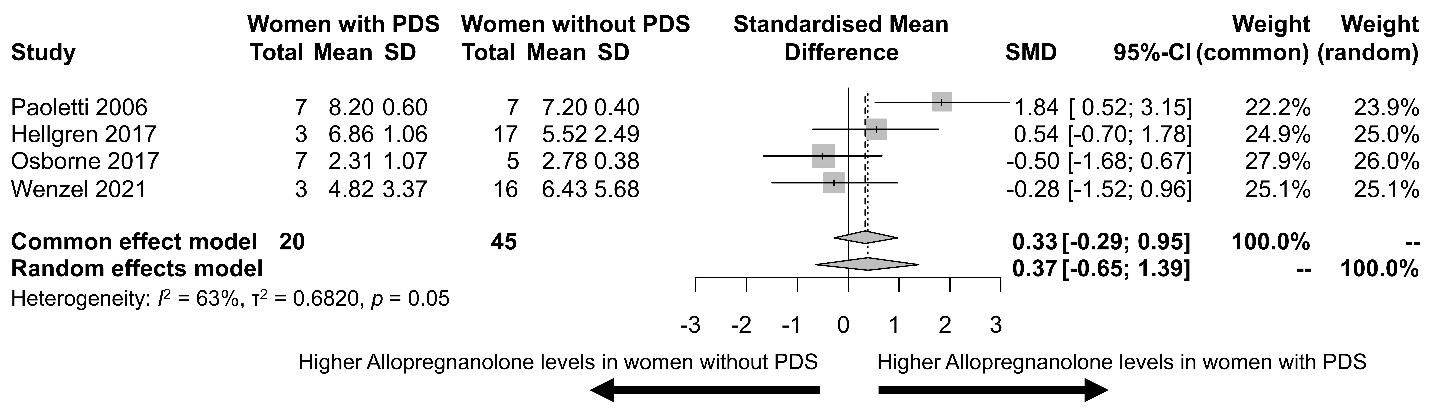


# **Supplementary Figure 3.** Allopregnanolone blood levels (ng/mL) in women with vs. without peripartum depressive symptoms (PDS) at gestational weeks 17-20. CI: confidence interval; SD: standard deviation; SMD: standardized mean difference


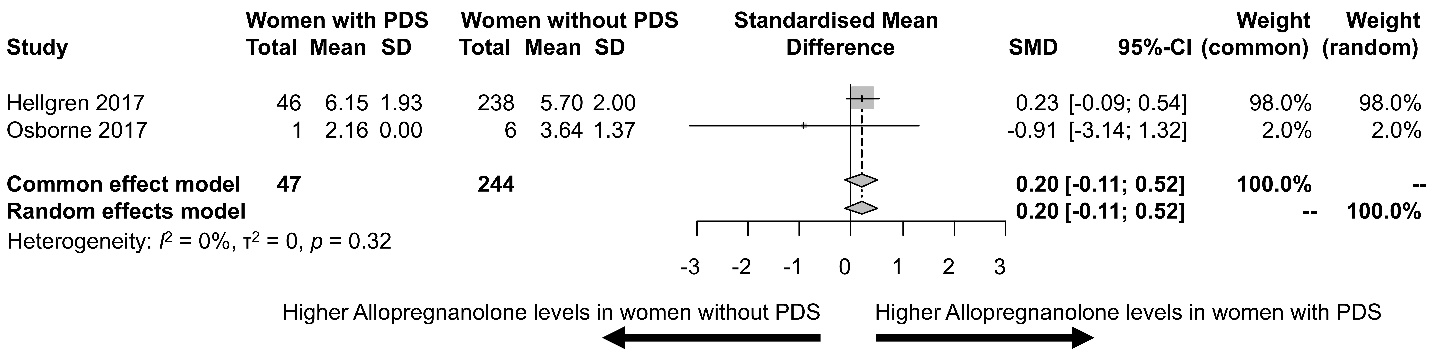


# **Supplementary Figure 4.** Allopregnanolone blood levels (ng/mL) in women with vs. without peripartum depressive symptoms (PDS) at gestational weeks 21-24. CI: confidence interval; SD: standard deviation; SMD: standardized mean difference


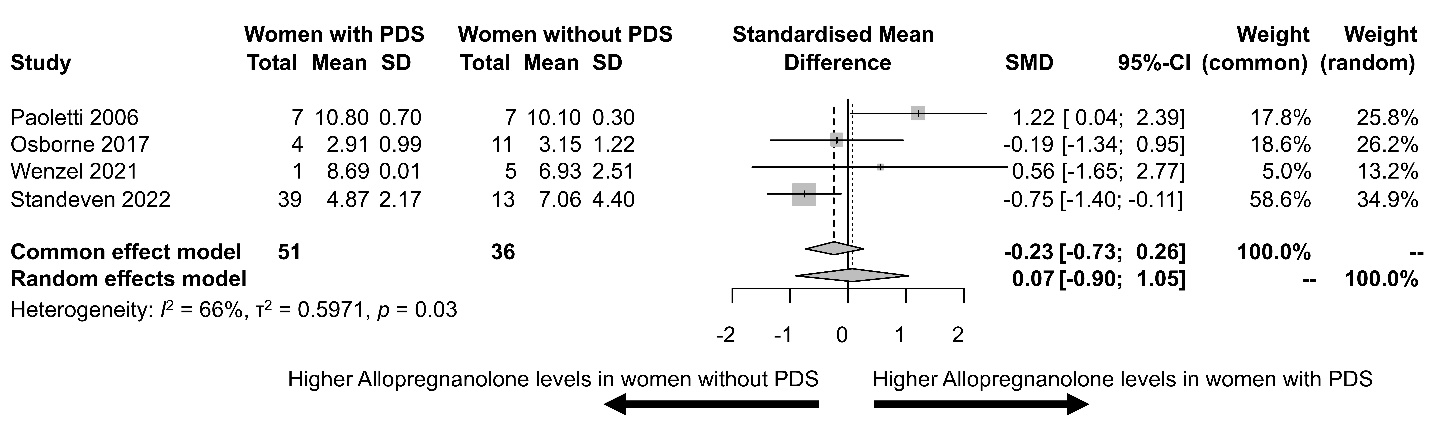


# **Supplementary Figure 5.** Allopregnanolone blood levels (ng/mL) in women with vs. without peripartum depressive symptoms (PDS) at gestational weeks 25-28. CI: confidence interval; SD: standard deviation; SMD: standardized mean difference


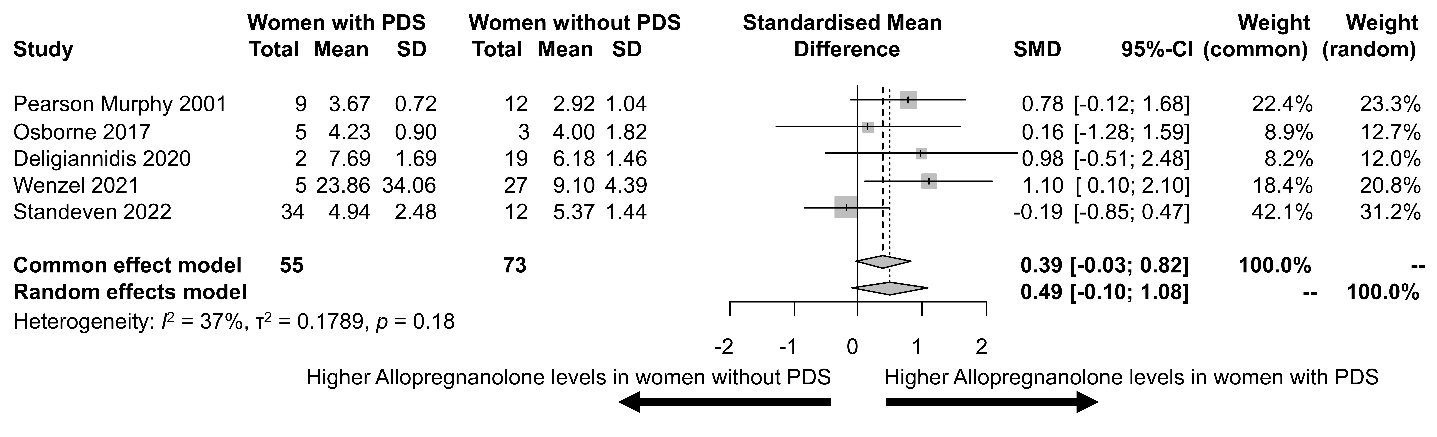


# **Supplementary Figure 6.** Allopregnanolone blood levels (ng/mL) in women with vs. without peripartum depressive symptoms (PDS) at gestational weeks 29-33. CI: confidence interval; SD: standard deviation; SMD: standardized mean difference


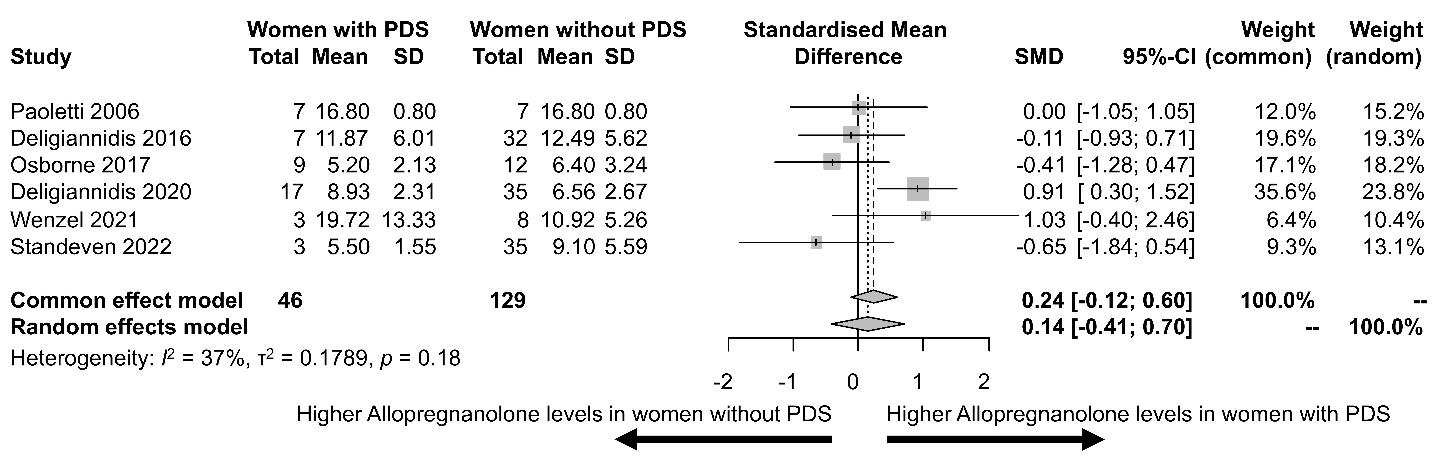


# **Supplementary Figure 7.** Allopregnanolone blood levels (ng/mL) in women with vs. without peripartum depressive symptoms (PDS) at ≥34 gestational weeks. CI: confidence interval; SD: standard deviation; SMD: standardized mean difference


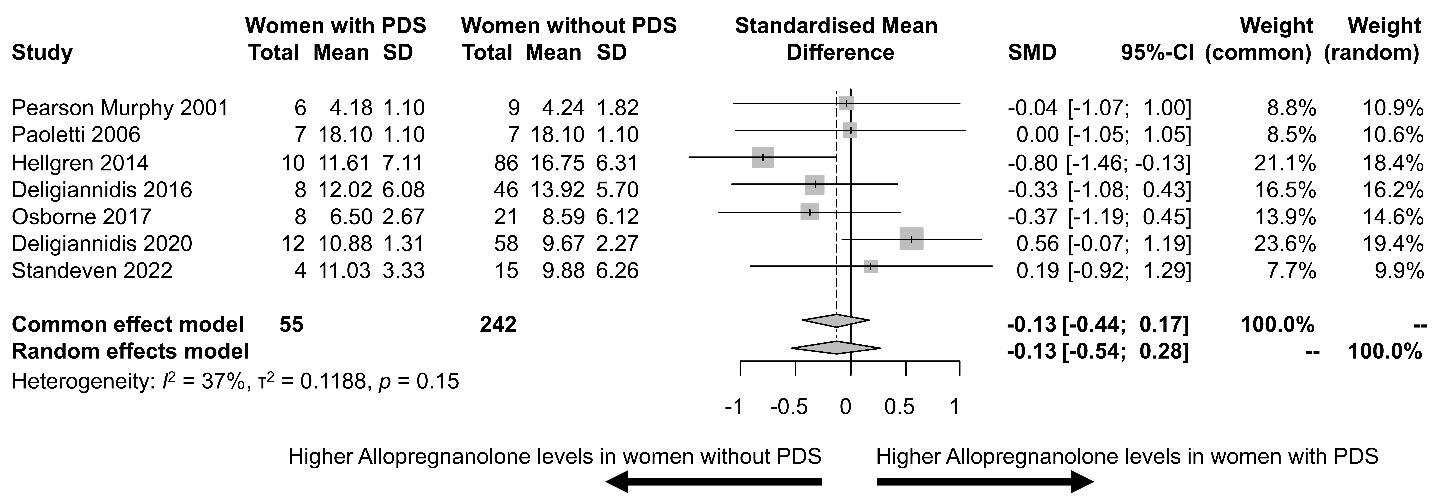


# **Supplementary Figure 8.** Allopregnanolone blood levels (ng/mL) in women with vs. without peripartum depressive symptoms (PDS) at ≤1 week postpartum. CI: confidence interval; SD: standard deviation; SMD: standardized mean difference


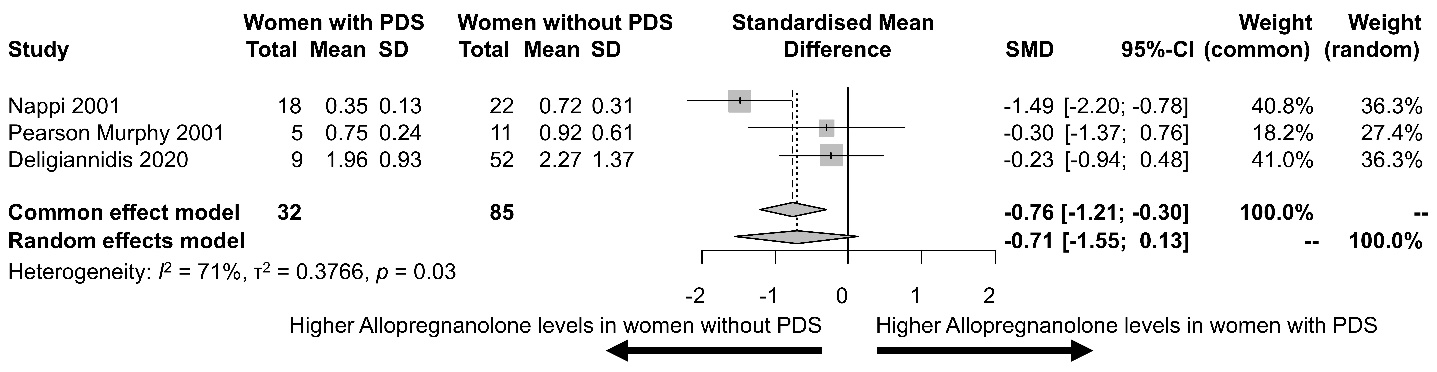


# **Supplementary Figure 9.** Allopregnanolone blood levels (ng/mL) in women with vs. without peripartum depressive symptoms (PDS) ≥2 weeks at postpartum. CI: confidence interval; SD: standard deviation; SMD: standardized mean difference


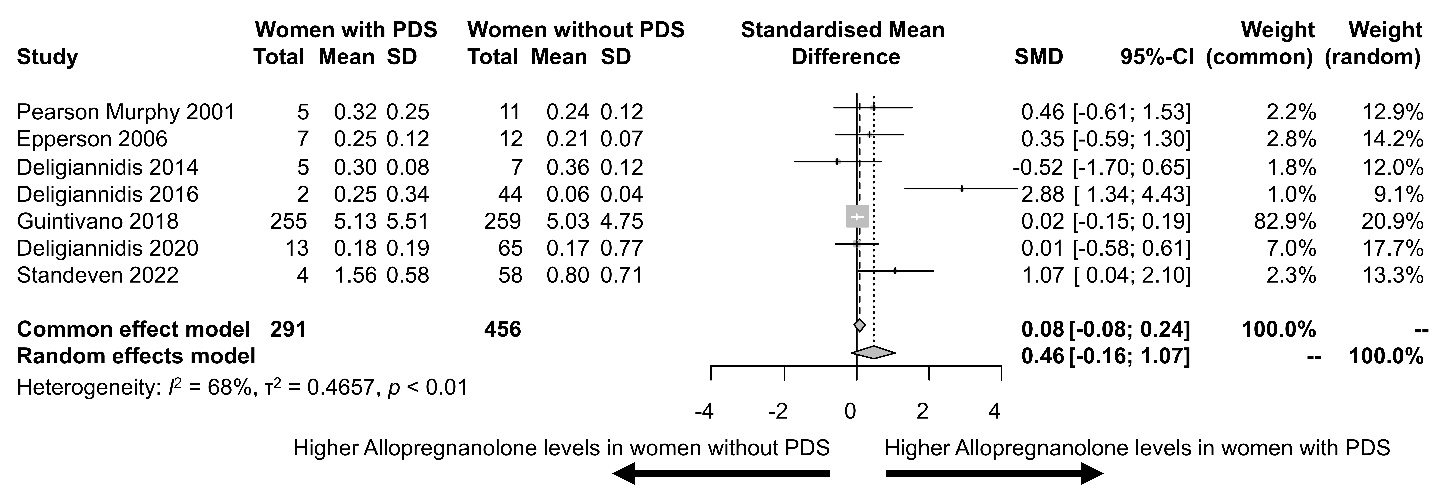


# **Supplementary Table 1.** Quality of included studies

| **Study**  **(Author, year)** | **Quality of studies** | | | |
| --- | --- | --- | --- | --- |
|  | **Selection (0-5)** | **Comparability (0-2)** | **Outcome (0-2)** | **Overall Judgement** |
| Nappi, 2001 | 2 | 2 | 0 | Poor |
| Pearson Murphy, 2001 | 2 | 1 | 1 | Fair |
| Epperson, 2006 | 2 | 2 | 1 | Fair |
| Paoletti, 2006 | 0 | 1 | 1 | Poor |
| Deligiannidis, 2013 | 3 | 2 | 2 | Good |
| Hellgren, 2014 | 3 | 2 | 1 | Fair |
| Deligiannidis, 2016 | 2 | 2 | 1 | Fair |
| Hellgren, 2017 | 2 | 1 | 2 | Fair |
| Osborne, 2017 | 1 | 2 | 1 | Fair |
| Guintivano, 2018 | 2 | 2 | 1 | Fair |
| Deligiannidis, 2020 | 3 | 2 | 1 | Fair |
| Wenzel, 2021 | 4 | 1 | 1 | Fair |
| Standeven, 2022 | 2 | 1 | 1 | Fair |
